# *Porphyromonas gingivalis* lipopolysaccharide regulates ephrin/Eph signalling in human periodontal ligament fibroblasts

**DOI:** 10.1111/jre.12463

**Published:** 2017-06-07

**Authors:** M. Li, C. Zhang, L. Jin, K. Matsuo, Y. Yang

**Affiliations:** ^1^ Faculty of Dentistry University of Hong Kong Hong Kong China; ^2^ Keio University School of Medicine Tokyo Japan

**Keywords:** ephrin/Eph, lipopolysaccharide, periodontal ligament fibroblasts

## Abstract

**Objective:**

EphrinA2‐EphA2 and ephrinB2‐EphB4 critically engage in bidirectional signalling to modulate alveolar bone remodelling. The present study aimed to investigate the effects of lipopolysaccharides (LPS) derived from *Porphyromonas gingivalis* on ephrin/Eph signalling in periodontal ligament fibroblasts (PDLFs).

**Material and Methods:**

The primary cultured PDLFs were incubated in the absence (as a control) or presence of *P. gingivalis* LPS at 0.001‐10 μg/mL for 24 hours. The PDLFs were then stimulated with *P. gingivalis* LPS at the optimal concentration (0.1 μg/mL) for different periods (6‐48 hours). The expression of ephrinA2, ephrinB2, EphA2 and EphB4 was assessed by quantitative reverse‐transcription real‐time polymerase chain reaction and western blotting. The osteoblastic markers alkaline phosphatase, osteocalcin and Runt‐related transcription factor 2 (Runx2), and the osteoclastogenesis‐related factors receptor activator of nuclear factor kappa‐B ligand (RANKL) and osteoprotegerin were also evaluated.

**Results:**

The ephrinA2 and EphA2 expression was upregulated and EphB4 expression was downregulated by stimulation of *P. gingivalis* LPS. EphrinA2 mRNA expression in the PDLFs was significantly upregulated from 12 to 48 hours (*P*<.05), whereas EphA2 exhibited no change for the first 24 hours, after which there was a significant increase at 48 hours (*P*<.05). EphB4 exhibited lower mRNA expression at 12 and 24 hours than did the control (*P*<.05), but the change was insignificant at 48 hours. In contrast, the expression of ephrinB2 remained unchanged. The expressions of ephrinA2, EphA2, ephrinB2 and EphB4 at the protein level showed a similar pattern to that at the mRNA level. The expression of Runx2 and osteocalcin significantly decreased, whereas that of RANKL/osteoprotegerin increased.

**Conclusion:**

The present study suggest that *P. gingivalis* LPS would contribute to a dysregulation of bone remodelling, whereby ephrinA2/EphA2 expression is stimulated and EphB4 expression is inhibited.

## 1 INTRODUCTION

The periodontium, a specialized tissue that surrounds the tooth root, is composed of the gingiva, the periodontal ligament (PDL), cementum and alveolar bone. As a part of the soft tissue in the periodontium, the PDL is a group of highly cellular, fibrous and connective tissues that attaches the teeth to the surrounding alveolar bone via the cementum. It is reported that the PDL has the capacity to secrete the biochemical substances involved in the regulation of alveolar bone remodelling.^1^ In the mechanism of bone‐related activity, Runt‐related transcription factor 2 (Runx2) is associated with osteoblast differentiation.^2^ Alkaline phosphatase (ALP) is closely associated with bone mineralization, and together with osteocalcin, acts as a hallmark of osteoblast differentiation in the regulation of osteogenesis. In contrast, the receptor activator of nuclear factor kappa‐B ligand (RANKL)‐osteoprotegerin (OPG) axis is known to be a master factor regulating osteoclast formation.^3^ Under this physiological condition, the maintenance of alveolar bone is achieved through a dynamic equilibrium between bone deposition and resorption. However, the balance of this process is disturbed by the presence of chronic periodontal inflammation, resulting in activation of osteoclastogenic factors and subsequent bone loss.^4^

Recent research has found that bone cell interactions are modulated by bidirectional signalling, ephrin ligands and Eph receptors.^5^ The receptor tyrosine kinases of the Eph family interact with the ephrin ligands at the cell surface because both are transmembrane proteins.^6^ Eph and ephrin molecules function as a receptor‐ligand pair in cell‐cell communication processes, triggering bidirectional signal transduction; namely, forward signalling via Ephs and reverse signalling via ephrin ligands.^7^ Both ephrins and Ephs are divided into two subclasses, A and B. Recent studies have indicated that various bone cells express ephrin ligands and Eph receptors, of which ephrinB2‐EphB4 and ephrinA2‐EphA2 differentially regulate the bone biology.^8^, ^9^ In that way, the bidirectional signalling between osteoclastic ephrinB2 and osteoblastic EphB4 suppresses osteoclastogenesis and enhances osteogenesis in osteoclast‐osteoblast communication. In contrast, reverse signalling via ephrinA2 and forward signalling via EphA2 reinforce osteoclastic bone resorption and inhibit osteoblastic bone formation.^5^ Studies on ehprin‐Eph largely focused on bone cells until 2011, when Diercke et al.^10^, ^11^ reported that periodontal ligament fibroblasts (PDLFs) also express ephrin/Eph molecules. Their research also revealed that ephrinB2‐EphB4 and ephrinA2‐EphA2 signalling are particularly important for the regulation of bone remodelling during orthodontic force loading between PDLFs and osteoblasts. Beyond the aforementioned studies by Diercke et al., little research has elaborated on the ephrin/Eph signalling in PDL and alveolar bone. Therefore, further investigation of this bidirectional signalling in PDL is worthwhile, particularly its role in periodontal inflammation when the bone remodelling balance has been broken and the osteoclastogenic factors and subsequent bone loss are activated. The results are expected to clarify the mechanism and shed light on the prevention and treatment of periodontal inflammation diseases.

It is well established that periodontitis, or inflammation of the periodontium, is triggered by pathogenic bacteria (mainly gram‐negative anaerobes such as *Porphyromonas gingivalis* 12 in the biofilm. It is characterized by the pathologic destruction of tooth‐supporting tissues, which can cause loss of the tooth. Lipopolysaccharides (LPS), the major element in the outer membranes of gram‐negative bacteria, act as prototypical endotoxins. LPS derived from *P. gingivalis* have been shown to promote the secretion of periodontal disease‐related proinflammatory cytokines by PDLFs.^13^ LPS are also involved in RANKL and OPG‐regulated osteoclast formation and bone resorption in PDLFs,^14^ and their inhibitory effect on the osteoblastic differentiation of osteoprogenitor cells.^15^ LPS are thus believed important pathogens in periodontitis that can lead to alveolar bone resorption in most advanced cases. Although LPS have been extensively studied and shown to contribute to the pathogenesis of periodontal disease,^16^ their possible effects on periodontitis bone resorption via ephrin/Eph signalling are still poorly understood. PDLFs are the dominant cell population in the PDL, and have been shown to express high levels of ALP activity and to secrete RANKL and OPG, indicating that they participate in bone remodelling.^17^ Therefore, it has been argued that the PDL plays a crucial role in alveolar bone metabolism. Collectively, we hypothesized that ephrin/Eph expression is also regulated by LPS stimuli in PDLFs. This study was therefore designed to extend the roles of the ephrinB2‐EphB4 and ephrinA2‐EphA2 systems to include PDLFs during inflammatory events.

## 2 MATERIAL AND METHODS

### 2.1 Primary cell culture

Human PDLFs were cultured using the method described in earlier research with slight modifications.^18^ Healthy premolars extracted for orthodontic purposes were collected from four healthy donors (aged 13.5±1.7 years) who gave signed informed consent according to the Institutional Review Board of the University of Hong Kong (IRB: UW13‐120). Briefly, to obtain primary PDLF tissue fragments of the PDL were isolated from the middle third surface of the tooth root with a scalpel. The cells were cultured in modified Eagle's medium‐alpha, supplemented with 10% foetal bovine serum and 1% antibiotics (100 U/mL penicillin and 100 μg/mL streptomycin) and incubated at 37°C in a humidified atmosphere with 5% CO_2_. The culture medium was replaced every 3 days after passaging, and the fifth passage cells cultured in monolayers were ready to be used for all experiments.

### 2.2 Lipopolysaccharide challenge of periodontal ligament fibroblasts

After reaching confluence, the PDLFs were seeded at a density of 5×10^4^ cells per well in a six‐well plate. At 80% confluence, the cells were starved for 12 hours in modified Eagle's medium alpha without foetal bovine serum and then treated with ultrapure LPS derived from *P. gingivalis* (InvivoGen, San Diego, CA, USA). To test the dose‐dependent effects, the PDLFs were cultured in the presence of *P. gingivalis* LPS at concentrations of 0.001, 0.01, 0.1, 1 and 10 μg/mL for 24 hours. The concentrations of *P. gingivalis* LPS (0.001‐10 μg/mL) we selected, which could be refined to different experiment protocols, are based on previous research regarding the in‐vitro PDLF cultures.^19^, ^20^, ^21^ To investigate the time‐dependent effects, *P. gingivalis* LPS of the optimal concentration (0.1 μg/mL) was used to stimulate the PDLFs for different experimental periods (6, 12, 24 and 48 hours). Untreated PDLFs served as the control. The heterogeneity of *P. gingivalis* LPS lipid A structure can be influenced by hemin concentration and temperature,^22^, ^23^ thus presents two different isoforms including PgLPS_1690_ and PgLPS_1435/1449_ that differentially mediate the expression of immuno‐inflammatory cytokines.^24^, ^25^ The *P. gingivalis* LPS used in the present study (LPS‐PG Ultrapure, InvivoGen Catalogue number: tlrl‐ppglps) is a highly purified preparation of LPS from the gram‐negative bacteria *P. gingivalis*, which is a generally used one obtained from InvivoGen and used in other studies.^26^, ^27^, ^28^ However, the isoform and molecular weight of the *P. gingivalis* LPS have not been specified by the manufacturer.

### 2.3 Quantitative reverse‐transcription real‐time polymerase chain reaction

Total cellular messenger RNA (mRNA) was extracted from the PDLFs using an RNeasy Mini Kit (Qiagen, Hilden, Germany), and its concentration was determined by the absorbance measurement at 260 nm. Complementary DNA (cDNA) was then synthesized from the mRNA using the SuperScript III First‐Strand Synthesis System (Invitrogen, Carlsbad, CA, USA). The concentration of mRNA used for quantitative reverse transcription‐polymerase chain reaction (qRT‐PCR) was 80‐150 ng/μL. The concentration of cDNA after RT was diluted in a 1:4 ratio. To analyse the relative gene expression level of the target genes, qRT‐PCR was performed with SYBR Green as a fluorescent dye in a StepOnePlus Real‐Time PCR System (Applied Biosystems, Carlsbad, CA, USA). Glyceraldehyde‐3‐phosphate dehydrogenase was used as the house‐keeping gene for endogenous control and for calculating the target gene expression. The expressions of ephrin B2, EphB4, ephrinA2 and EphA2 were assessed. To gain a more detailed insight into the mechanism of LPS‐induced bone remodelling at the molecular level, the expression of some osteoblast differentiation genes (Runx2, ALP and osteocalcin) and osteoclastogenesis‐related genes (RANKL and OPG) in PDLFs were also monitored using qRT‐PCR after a 24 hour challenge with LPS (0.1 μg/mL). All of the primer sequences for the target genes are listed in Table 1. The amplification reactions were implemented in 96‐well plates with a mixture volume of 20 μL that contained 1 μL cDNA and 10 μL Power SYBR Green PCR Master Mix (Applied Biosystems) in each well with the following programme: initial heating to 95°C for 10 minutes, followed by 40 cycles of denaturation at 95°C for 15 seconds and annealing and extension at 60°C for 1 minutes.

**Table 1:** Primer sequences of the target genes for quantitative reverse transcription‐polymerase chain reaction

Gene name	Forward sequence (5′→3′)	Reverse sequence (5′→3′)	Sequence ID	Primer location (Fw‐Rv)
ephrinA2	CAATAAGCTCGTGTCGTCTGTCA	TAGTGGTTCCCTCCCTTTTGTC	NM_001405.3	1725‐1824
EphA2	TCAAGGACCAGGTGAACACTGT	AGGGAGGCCACTCTGTTTCTTC	NM_004431.3	3049‐3148
ephrinB2	TCCCTGGTCACCCGACTTT	ACTGTAACACCCCAAATCCATAGAC	NM_004093.3	3780‐3879
EphB4	GTGCCTTGGTCATCCCACAT	ACCAACTACCGCCCTTTTCA	NM_004444.4	4141‐4240
RANKL	AAAGCTGACATTGCCAAAAAGG	TTTGCGGCACTTGTGGAA	NM_003701.3	1938‐2037
OPG	GCCCTGACCACTACTACACAGACA	TTGCACTCCTGCTTGACGTACT	NM_002546.3	517‐616
Runx2	ACTGGCGCTGCAACAAGAC	TCATCGTTACCCGCCATGA	NM_001024630.3	596‐695
OCN	CGGTGCAGAGTCCAGCAAA	GGCTCCCAGCCATTGATACA	NM_199173.5	348‐447
ALP	CTGATGTGGAGTATGAGAGTGAC	CAGATGAAGTGGGAGTGCTTGT	NM_000478.5	959‐1073
GAPDH	TCCCTGAGCTGAACGGGAAG	GGAGGAGTGGGTGTCGCTGT	NM_002046.5	850‐1067

Annotations for target genes: ALP, alkaline phosphatase; Fw‐Rv, forward‐reverse; GAPDH, glyceraldehyde‐3‐phosphate dehydrogenase; OCN, osteocalcin; OPG, osteoprotegerin; RANKL, receptor activator of nuclear factor kappa‐B ligand; Runx2, runt‐related transcription factor 2.

### 2.4 Western blotting

The PDLFs were collected using an RIPA buffer (Thermo Scientific, Rockford, IL, USA) containing a protease inhibitor cocktail. The Pierce BCA Protein Assay Kit (Thermo Scientific) was used for total protein quantification. A lysate with 25 μg (for ephrinB2 and ephrinA2 detection), 30 μg (for EphA2 detection) or 35 μg (for EphB4 detection) of protein was then fractionated on 10% sodium dodecyl sulphate‐polyacrylamide gel and transferred on to a nitrocellulose membrane. The membranes were blocked for 1 hour at room temperature with 5% non‐fat milk in TBS‐T (10 mM Tris, 100 mM NaCl and 0.1% Tween20), after which they were probed overnight at 4°C with rabbit anti‐ephrinB2 monoclonal antibody (1:1000; Abcam, Cambridge, UK), rabbit anti‐EphB4 monoclonal antibody (1:500; Cell Signaling Technology, Danvers, MA, USA), rabbit ephrinA2 polyclonal antibody (1:500; Abcam) or rabbit anti‐EphA2 monoclonal antibody (1:1000; Cell Signaling Technology). β‐Actin (1:1000; Cell Signaling Technology) served as the internal control. The densities of western blotting bands were measured by ImageJ (an open source Java image processing program inspired by NIH Image, https://imagej.nih.gov/ij/).

### 2.5 Statistical analysis

The experiments were performed in triplicate and the results were expressed as means±standard deviation (SD). The data were compared with independent two‐tailed *t* tests. A *P* value of .05 was considered to show statistical significance.

## 3 RESULTS

### 3.1 mRNA expressions of ephrinB2‐EphB4 and ephrinA2‐EphA2 in a *P. gingivalis* lipopolysaccharide dose‐dependent manner

**Figure 1:**
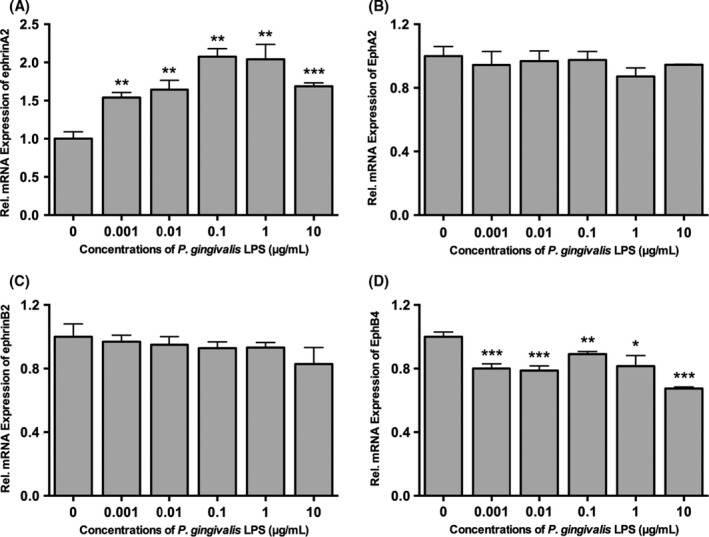
Dose‐dependent effects of *Porphyromonas gingivalis* LPS on ephrinA2‐EphA2 and ephrinB2‐EphB4 mRNA expression in periodontal ligament fibroblasts determined by quantitative reverse transcription‐polymerase chain reaction. Expression of ephrinA2 (A), EphA2 (B), ephrinB2 (C) and EphB4 (D) in LPS‐stimulated periodontal ligament fibroblasts when cells were treated with *P. gingivalis* LPS at five different concentrations (0.001, 0.01, 0.1, 1 and 10 μg/mL) for 24 h. Data were presented as mean±SD. *P<.05, **P<.01, ***P<.001 vs control. LPS, lipopolysaccharide

After culturing with *P. gingivalis* LPS at different concentrations for 24 hours, the mRNA expression levels of ephrinB2‐EphB4 and ephrinA2‐EphA2 in the PDLFs were assessed. EphB4 expression exhibited a significant downward trend (*P*<.05) with unaltered ephrinB2 expression, whereas ephrinA2 was significantly up‐regulated (*P*<.05) and accompanied by unaffected EphA2 in LPS‐stimulated PDLFs. The results of the qPCR showed no striking dose‐dependent relationship among these changes, except that the expression of ephrinA2 reached its peak when the *P. gingivalis* LPS concentration was 0.1 μg/mL (*P*<.01) (Figure 1). A *P. gingivalis* LPS concentration of 0.1 μg/mL was therefore considered optimal for the subsequent experiments.

### 3.2 Time‐dependent mRNA Expression of ephrinB2‐EphB4 and ephrinA2‐EphA2

**Figure 2:**
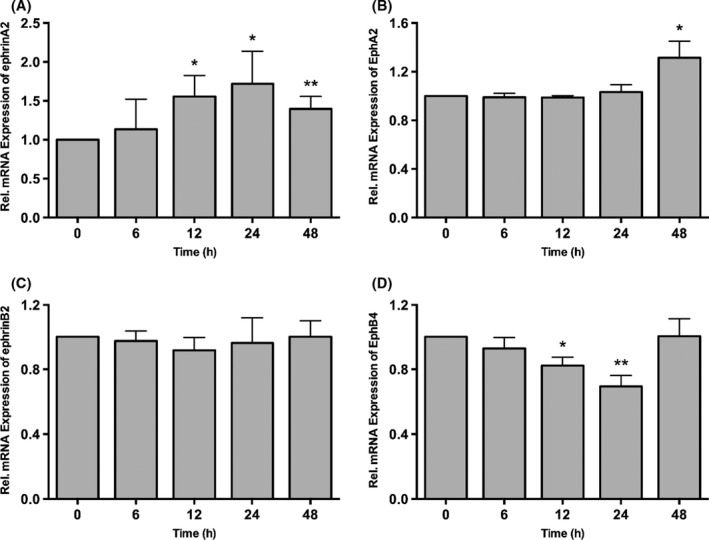
Time‐dependent effects of *Porphyromonas gingivalis* LPS on ephrinA2‐EphA2 and ephrinB2‐EphB4 mRNA expression in periodontal ligament fibroblasts determined by quantitative reverse transcription‐polymerase chain reaction. Expression of ephrinA2 (A) and EphA2 (B), ephrinB2 (C) and EphB4 (D) in LPS‐stimulated periodontal ligament fibroblasts when cells were treated with 0.1 μg/mL *P. gingivalis* LPS for 6, 12, 24 and 48 h. Data were presented as mean±SD. *P<.05, **P<.01 vs control. LPS, lipopolysaccharide

EphB4 expressed lower levels of mRNA at 12 hours (*P*<.05) and 24 hours (*P*<.01) than did the control group, but the change was insignificant at 48 hours (Figure 2D). The expression of ephrinB2 remained unchanged with different concentrations of *P. gingivalis* LPS over the treatment time (Figures 1C and 2C).

EphrinA2 expression significantly increased in a time‐dependent manner from 12 to 24 hours (*P*<.05) and slightly decreased at 48 hours, but remained markedly higher than in the control group (*P*<.01) (Figure 2A). The expression of EphA2 showed no change over the first 24 hours, after which a significant increase was seen at 48 hours (*P*<.05) (Figure 2B).

### 3.3 Expression of ephrinB2‐EphB4 and ephrinA2‐EphA2 at the protein level

**Figure 3:**
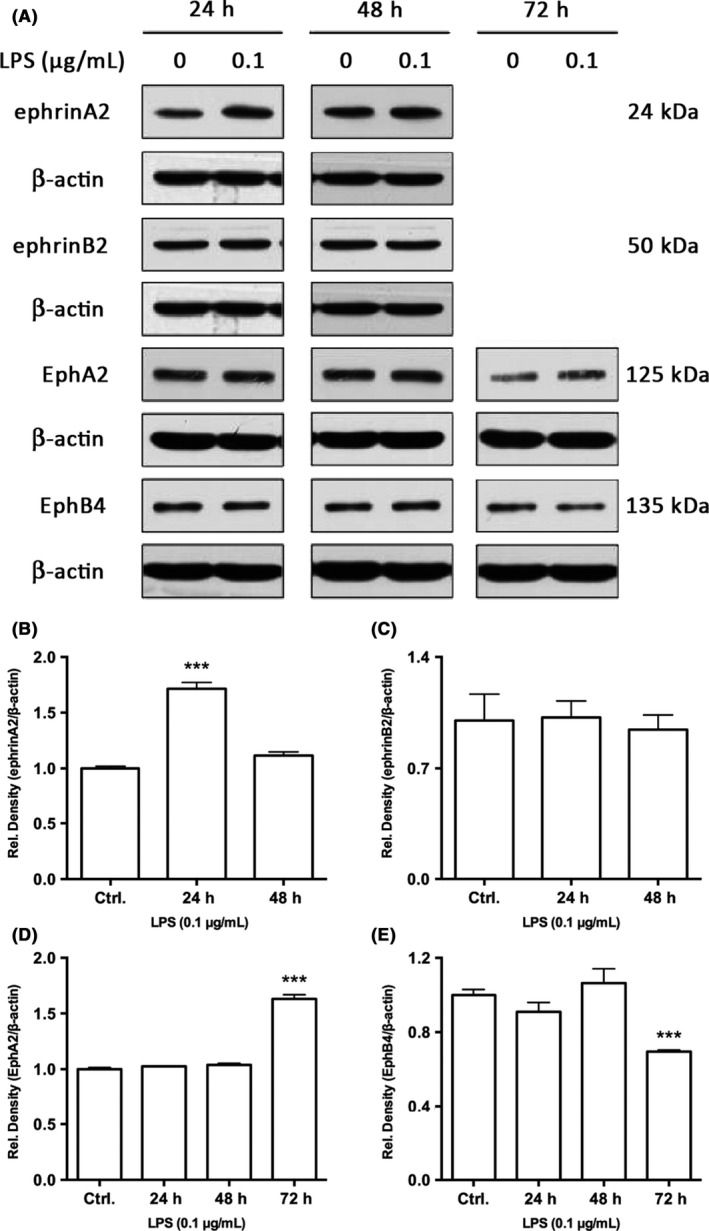
Protein expression of ephrinA2‐EphA2 and ephrinB2‐EphB4 (A) in periodontal ligament fibroblasts stimulated with 0.1 μg/mL *Porphyromonas gingivalis* LPS as determined by western blot. Band densities (B‐E) were measured by ImageJ software. Data were presented as mean±SD. ***P<.001 vs control. LPS, lipopolysaccharide

The results of western blotting confirmed the findings of the mRNA expression level. The changing tendency at the mRNA and protein levels was the same but was observed at different treatment time points. The protein expressions of ephrinA2 and EphA2 were increased at 24 and 72 hours, respectively (Figure 3), whereas the upregulation of ephrinA2 mRNA expression was found from 12 to 48 hours and EphA2 was upregulated at 72 hours (Figure 2B). The downregulation of EphB4 protein expression was not observed until 72 hours (Figure 3), whereas its mRNA expression decreased from 12 to 24 hours after stimulation with *P. gingivalis* LPS (Figure 2D). Similar to the result at the mRNA level, ephrinB2 expression showed no significant difference at the protein level (Figure 3). In particular, the protein expression was first measured for 24 and 48 hours, yet the level of EphA2 and EphB4 had no significant changes during these periods. Assuming that the post‐transcription modification and translation process may affect the timing of mRNA and protein expression, we extend the treatment time to 72 hours. As expected, both the expressions of EphA2 and EphB4 were found the same varying tendency as the gene expression did. As the protein expression of ephrinA2 went down to the control level again after 48 hours of stimulation, and that of ephrinB2 was unchanged at the mRNA level from 24 to 48 hours, their expression at protein levels within 48 hours has confirmed that at mRNA levels, thus the protein expression of ephrinA2 and ephrinB2 after 48 hours were not further assessed.

### 3.4 Expression of osteoblastic markers (alkaline phosphatase, osteocalcin and Runt‐related transcription factor 2) and osteoclastogenesis‐related factors (receptor activator of nuclear factor kappa‐B ligand and osteoprotegerin)

**Figure 4:**
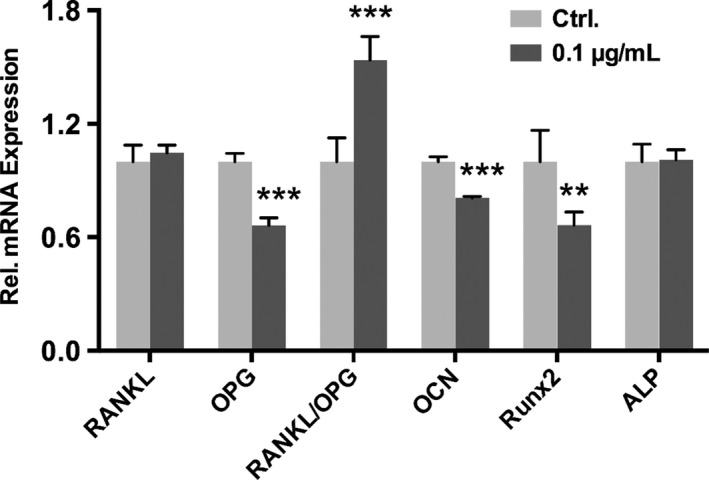
Effects of 0.1 μg/mL *Porphyromonas gingivalis* lipopolysaccharide on the expression of osteoclastogenesis‐related factors (RANKL and OPG) and osteoblastic markers (ALP, OCN and Runx2) in periodontal ligament fibroblasts determined by quantitative reverse transcription‐polymerase chain reaction when cells were treated with 0.1 μg/mL *P. gingivalis* lipopolysaccharide for 24 h. Data were presented as mean±SD. **P<.01, ***P<.001 vs control. ALP, alkaline phosphatase; OCN, osteocalcin; OPG, osteoprotegerin; RANKL, receptor activator of nuclear factor kappa‐B ligand; Runx2, runt‐related transcription factor 2

For the osteoblastic markers, the PDLFs expressed decreasing mRNA of Runx2 (*P*<.01) and osteocalcin (*P*<.001) after stimulation with 0.1 μg/mL *P. gingivalis* LPS for 24 hours. No significant variation in ALP was found (Figure 4). In contrast, the RANKL/OPG ratio was upregulated significantly (*P*<.001) with decreased OPG (*P*<.001).

## 4 DISCUSSION

In the present study, we investigated the ephrin/Eph expression regulated by LPS stimuli in human PDLFs. It is known that periodontitis is caused by bacterial colonies and their metabolites, particularly LPS, accompanied by a host‐mediated immune response against these microorganisms and a progressive loss of alveolar bone. Accumulating evidence indicates that ephrins and Ephs affect bone homeostasis both in normal conditions and in a range of pathologic conditions such as osteoporosis and cancer‐induced bone disease.^29^ Although some studies have assessed through the regulation on ephrin/Eph signalling that LPS are involved,^30^, ^31^ they have largely evaluated the lung injury model^30^ or osteoblast‐osteoclast co‐culture systems.^31^ The regulation on ephrin/Eph signalling in human PDL with LPS stimulation has not been assessed. Therefore, in this study, we investigated the dose‐ and time‐dependent effects of *P. gingivalis* LPS on human PDL fibroblasts by evaluating ephrinB2‐EphB4 and ephrinA2‐EphA2 expression related to pathologic bone resorption. We found that *P. gingivalis* LPS enhanced ephrinA2 and EphA2 expression and attenuated EphB4 expression in PDLFs. Besides, several studies evaluated the in‐vivo amount of LPS in the case of periodontal disease, eg, the serum LPS concentration is approximately 1.0 ng/mL in patients with severe periodontitis.^32^ Another example^33^ has shown about 0.4 endotoxin unit/mL of LPS in the gingival crevicular fluid of a periodontitis rat model. However, these data are not comparable to the LPS dose used in in‐vitro studies as the LPS contained in the serum is pretty low and that measured in gingival crevicular fluid is presented with the activity of the endotoxin. Thus, we referred to the corresponding in‐vitro research^19^, ^20^, ^21^ and chose the most commonly used quantities of *P. gingivalis* LPS for our experiment planning.

The results of the qPCR revealed no striking dose‐dependent relationship among these changes, except that the expression of ephrinA2 reached its peak when the *P. gingivalis* LPS concentration was 0.1 μg/mL. We further treated the cells with 0.1 μg/mL *P. gingivalis* LPS from 6 to 48 hours. It is interesting to find that the upregulation of EphA2 (48 hours) lagged behind that of ephrinA2 (from 12 to 48 hours) at the transcriptional level, which suggests a disruption of the ephrinA2‐EphA2 balance. The bidirectional signalling between ephrins and Ephs is a result of the activation of different signalling pathways in both ligand‐ and receptor‐expressing cells.^29^ Class A ephrin/Eph families are also expressed in various bone cells and regulate bone remodelling. For example, EphA4 may contribute to ossification by functioning in osteoblasts and hypertrophic chondrocytes.^34^ EphA4 and EphA2 are both potential receptors for ephrinA2, but EphA2 is likely to be preferred among osteoclast precursors during early differentiation.^9^ In this case, the ephrinA2‐EphA2 interaction facilitates the initiation phase of bone remodelling by enhancing osteoclast differentiation and suppressing osteoblast differentiation. Both reverse ephrinA2 signalling and forward EphA2 signalling enhance osteoclast differentiation, but it is suppressed only by forward signalling via EphA2.^9^ We found that reverse ephrinA2 signalling was promoted at an earlier time point in LPS‐stimulated PDLFs and was followed by forward EphA2 signalling at a later stage upon LPS challenge. We assume that the overexpression of ephrinA2 reverse signalling that increases bone resorption is initially promoted and then has a reciprocal interaction with the repression of bone formation via EphA2 forward signalling. Western blot analysis confirmed the LPS‐stimulated production of ephrinA2‐EphA2 in PDLFs.

**Figure 5:**
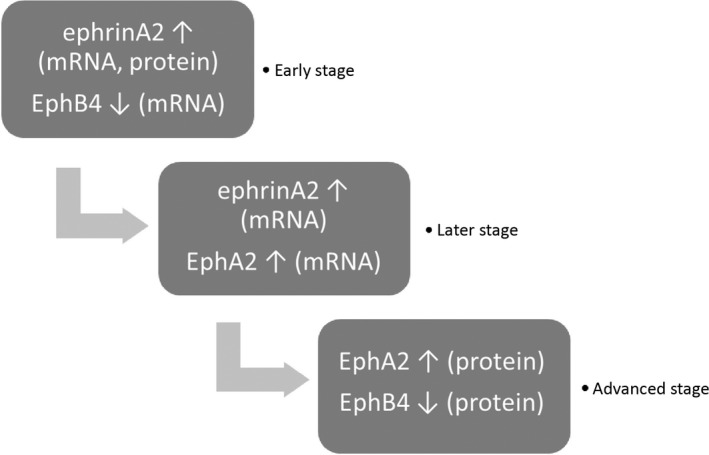
Variations of ephrin/Eph signalling in periodontal ligament fibroblasts upon *Porphyromonas gingivalis* lipopolysaccharide stimulation

In contrast, *P. gingivalis* LPS decreased EphB4 expression at the transcriptional level at 12 and 24 hours but had no significant effect on ephrinB2. The interaction of ephrinB2‐EphB4 related to bone remodelling is mainly investigated in the coupling communication between osteoclasts and osteoblasts. Zhao et al.^8^ proposed two‐way communication between the ephrinB2 ligand on osteoclasts and EphB4 receptor on osteoblasts, ie, generating bidirectional signalling when in contact with each other, ephrinB2 and EphB4 transmit biological signals into both ephrinB2‐expressing cells and EphB4‐expressing cells. Through an in‐vitro study, Zhao et al. found that forward signalling via EphB4 enhances osteoblast formation, whereas reverse signalling via ephrinB2 inhibits osteoclast formation. They also generated EphB4 transgenic mice, indicating that the overexpression of EphB4 in osteoblasts enhances bone formation and suppresses bone resorption in vivo. There is also evidence that blocking ephrinB2‐EphB4 interaction inhibits mineralization in osteoblasts.^35^ Tonna et al.^36^ even ascribed the influence of ephrinB2 in the promotion of osteoblast differentiation to its limiting effect on cell apoptosis. Bone homeostasis requires the activities of bone marrow‐derived mesenchymal stem cells, among which the bidirectional communication of ephrinB2 and EphB4 signalling has been reported to play a crucial role. For example, overexpression of ephrinB2 in mesenchymal stem cells displays enhanced osteogenesis and mineralization via its interaction with EphB4.^37^ Moreover, the ephrinB2‐stimulated forward signalling through EphB4 enhances osteogenic differentiation via cross‐talk with the Wnt pathway.^38^ Unlike ephrinB2, which interacts with multiple Ephs, EphB4 only binds to ephrinB2.^39^ That is to say, the decrease of cell surface EphB4 expression suggests suppression of forward signalling via EphB4 activities. Because EphB4 forward signalling is considered to stimulate osteoblast differentiation,^8^ its downregulation in PDLFs after treatment with *P. gingivalis* LPS may be associated with the inhibition of bone formation. Furthermore, the decreased transcriptional expression of EphB4 and the opposite change of ephrinA2 have a synergistic effect on bone resorption in the early stages. It is interesting that along with the insignificant change in EphB4 by 48 hours, the ephrinA2‐EphA2 interaction began to have a major effect on the regulation of bone homeostasis, as both ephrinA2 and EphA2 mRNA were upregulated by 48 hours. Figure 5 shows the variations of ephrin/Eph signalling in PDLFs upon *P. gingivalis* LPS stimulation. However, these data are not consistent with those of a previous study conducted in an osteoblast‐osteoclast co‐culture system treated with *P. gingivalis* LPS, which promoted EphB4 but attenuated ephrinB2 expression.^31^ Namely, in the co‐culture system, *P. gingivalis* LPS might contribute to the promotion of EphB4‐mediated osteoblast differentiation and the inhibition of ephrinB2‐related osteoclast differentiation. However, it seems that in PDLFs, *P. gingivalis* LPS exerts a negative role in osteoblastogenesis through decreasing EphB4 expression. In addition, this discrepancy may be due to the different behaviour between the PDL and alveolar bone. The protein expression of ephrinB2 and EphB4 showed a similar pattern with mRNA levels, although some differences were observed in the treatment time points; ie, the EphB4 protein level was suppressed in the later stages compared with the mRNA level, probably due to the lag between transcription and translation.

It has been reported that applying compressive force induces the expression of ephrinA2‐EphA2 and reduces that of ephrinB2‐EphB4 in PDLFs.^11^ The bone resorption on the compression side during orthodontic tooth movement is mediated by the ephrin/Eph system. In our research on LPS‐stimulated PDLFs, ephrinA2‐EphA2 and ephrinB2‐EphB4 showed changing trends similar to that in the study on compressive force,^11^ which suggests that ephrin/Eph can also be modulated by an inflammatory reaction and affects the regulation of bone remodelling.

To gain a more profound profile about the behaviour of PDLFs under inflammatory challenge, we also stimulated the cells with the optimal concentration (0.1 μg/mL) of *P. gingivalis* LPS for 24 hours, which is the same condition as the evaluation of ephrin/Eph signalling, and assessed the mRNA expression of osteogenic and osteoclastogenic genes. The interplay between RANKL and RANK is critical for osteoclast formation and can also enhance osteoclast activity. The OPG receptor, other than RANKL functioning as an osteoclast differentiation factor, is an osteoclastogenesis inhibitory factor that can also combine with RANK to prevent a RANK‐RANKL interaction and the consequent bone resorption. RANKL and OPG are related to periodontal disease, and a balance between them is essential in bone destruction processes.^40^ In this study, the ephrin/Eph system was modulated by *P. gingivalis* LPS challenge with a concurrent significant decrease in the RANKL/OPG ratio, which is in accordance with the report^14^ that the stimulation of PDLFs with LPS leads to increased RANKL expression. The results emphasize that ephrinA2‐EphA2 and ephrinB2‐EphB4 might exert a biological effect on LPS‐induced inflammatory bone destruction. The challenge of ephrinA2‐Fc has been demonstrated to downregulate the expression of Runx2 and ALP in PDLFs.^11^ Furthermore, EphB4 knockdown in osteoblasts displayed a lowered ALP activity.^8^ In the present study, the mRNA expression of Runx2 and osteocalcin was significantly downregulated, which suggests that the regulation of ephrins and Ephs in our setting might be responsible for the suppression of osteogenesis. It has been shown that the expression of Runx2 and osteocalcin in LPS‐treated osteoblasts is significantly suppressed,^41^ which is consistent with the present study.

To date, the *P. gingivalis* LPS has been demonstrated to regulate the expression of proinflammatory cytokines through activating toll‐like receptor (TLR) ‐2 or TLR‐4,^42^ while LPS from other gram‐negative bacteria is TLR‐1. The TLR‐2 activity of *P. gingivalis* LPS is ascribed to the result of lipoprotein contamination.^43^ The *P. gingivalis* LPS used in the present study was a commercial preparation purified by the supplier and has undergone enzymatic treatment to remove lipoproteins so that TLR‐2 is not activated according to the manufacturer's instructions. Whether TLR‐4 is dependent on the modulation of ephrin/Eph signalling needs further investigation in our future studies on the pathway regarding to ephrin/Eph signalling.

Positive regulation by means of enhancing the expression or negative regulation through attenuating the expression of ephrins or Ephs, and the determination of bone resorption activity, which is used to elucidate the functional consequences, were not performed in this study and will be investigated in a future study.In conclusion, the expression of ephrinA2‐EphA2 significantly increased and that of EphB4 decreased in PDLFs upon stimulation with *P. gingivalis* LPS. These changes occurred with a corresponding expressive variation in the osteoblast differentiation genes (Runx2 and osteocalcin) and osteoclastogenesis‐related genes (OPG and RANKL), indicating that ephrinA2‐EphA2 and ephrinB2‐EphB4 interaction might regulate inflammatory bone resorption. The results of this study will shed light on the development of therapeutic interventions on periodontitis.

## References

[1] Wada N , Maeda H , Tanabe K , et al. Periodontal ligament cells secrete the factor that inhibits osteoclastic differentiation and function: the factor is osteoprotegerin/osteoclastogenesis inhibitory factor. J Periodontal Res. 2001;36:56‐63. 11246705 10.1034/j.1600-0765.2001.00604.x

[2] Lian JB , Stein GS . Runx2/Cbfa1: a multifunctional regulator of bone formation. Curr Pharm Des. 2003;9:2677‐2685. 14529540 10.2174/1381612033453659

[3] Boyce BF , Xing L . Functions of RANKL/RANK/OPG in bone modeling and remodeling. Arch Biochem Biophys. 2008;473:139‐146. 18395508 10.1016/j.abb.2008.03.018 PMC2413418

[4] Sokos D , Everts V , de Vries TJ . Role of periodontal ligament fibroblasts in osteoclastogenesis: a review. J Periodontal Res. 2015;50:152‐159. 24862732 10.1111/jre.12197

[5] Matsuo K , Otaki N . Bone cell interactions through Eph/ephrin bone modeling, remodeling and associated diseases. Cell Adh Migr. 2012;6:148‐156. 22660185 10.4161/cam.20888 PMC3499314

[6] Kullander K , Klein R . Mechanisms and functions of Eph and ephrin signalling. Nat Rev Mol Cell Biol. 2002;3:475‐486. 12094214 10.1038/nrm856

[7] Pasquale EB . Eph‐ephrin bidirectional signaling in physiology and disease. Cell. 2008;133:38‐52. 18394988 10.1016/j.cell.2008.03.011

[8] Zhao C , Irie N , Takada Y , et al. Bidirectional ephrinB2‐EphB4 signaling controls bone homeostasis. Cell Metab. 2006;4:111‐121. 16890539 10.1016/j.cmet.2006.05.012

[9] Irie N , Takada Y , Watanabe Y , et al. Bidirectional signaling through ephrinA2‐EphA2 enhances osteoclastogenesis and suppresses osteoblastogenesis. J Biol Chem. 2009;284:14637‐14644. 19299512 10.1074/jbc.M807598200 PMC2682911

[10] Diercke K , Kohl A , Lux CJ , Erber R . Strain‐dependent up‐regulation of ephrin‐B2 protein in periodontal ligament fibroblasts contributes to osteogenesis during tooth movement. J Biol Chem. 2011;286:37651‐37664. 21880727 10.1074/jbc.M110.166900 PMC3199509

[11] Diercke K , Sen S , Kohl A , Lux CJ , Erber R . Compression‐dependent up‐regulation of ephrin‐A2 in PDL fibroblasts attenuates osteogenesis. J Dent Res. 2011;90:1108‐1115. 21724962 10.1177/0022034511413926

[12] Feng Z , Weinberg A . Role of bacteria in health and disease of periodontal tissues. Periodontol. 2000;2006:50‐76. 10.1111/j.1600-0757.2005.00148.x 16398685

[13] Morandini AC , Sipert CR , Gasparoto TH , et al. Differential production of macrophage inflammatory protein‐1alpha, stromal‐derived factor‐1, and IL‐6 by human cultured periodontal ligament and gingival fibroblasts challenged with lipopolysaccharide from P. gingivalis. J Periodontol. 2010;81:310‐317. 20151811 10.1902/jop.2009.090375

[14] Krajewski AC , Biessei J , Kunze M , Maersch S , Perabo L , Noack MJ . Influence of lipopolysaccharide and interleukin‐6 on RANKL and OPG expression and release in human periodontal ligament cells. APMIS. 2009;117:746‐754. 19775343 10.1111/j.1600-0463.2009.02532.x

[15] Kadono H , Kido J , Kataoka M , Yamauchi N , Nagata T . Inhibition of osteoblastic cell differentiation by lipopolysaccharide extract from Porphyromonas gingivalis. Infect Immun. 1999;67:2841‐2846. 10338489 10.1128/iai.67.6.2841-2846.1999 PMC96590

[16] Socransky SS , Haffajee AD . The bacterial etiology of destructive periodontal disease: current concepts. J Periodontol. 1992;63:322‐331. 1573546 10.1902/jop.1992.63.4s.322

[17] Jonsson D , Nebel D , Bratthall G , Nilsson BO . The human periodontal ligament cell: a fibroblast‐like cell acting as an immune cell. J Periodontal Res. 2011;46:153‐157. 21118418 10.1111/j.1600-0765.2010.01331.x

[18] Howard PS , Kucich U , Taliwal R , Korostoff JM . Mechanical forces alter extracellular matrix synthesis by human periodontal ligament fibroblasts. J Periodontal Res. 1998;33:500‐508. 9879524 10.1111/j.1600-0765.1998.tb02350.x

[19] Pumklin J , Bhalang K , Pavasant P . Hypoxia enhances the effect of lipopolysaccharide‐stimulated IL‐1 beta expression in human periodontal ligament cells. Odontology. 2016;104:338‐346. 26810902 10.1007/s10266-015-0223-4

[20] Wada N , Maeda H , Yoshimine Y , Akamine A . Lipopolysaccharide stimulates expression of osteoprotegerin and receptor activator of NF‐kappa B ligand in periodontal ligament fibroblasts through the induction of interleukin‐1 beta and tumor necrosis factor‐alpha. Bone. 2004;35:629‐635. 15336598 10.1016/j.bone.2004.04.023

[21] Yamaji Y , Kubota T , Sasaguri K , et al. Inflammatory cytokine gene‐expression in human periodontal‐ligament fibroblasts stimulated with bacterial lipopolysaccharides. Infect Immun. 1995;63:3576‐3581. 7642293 10.1128/iai.63.9.3576-3581.1995 PMC173496

[22] Al‐Qutub MN , Braham PH , Karimi‐Naser LM , Liu X , Genco CA , Darveau RP . Hemin‐dependent modulation of the lipid A structure of *Porphyromonas gingivalis* lipopolysaccharide. Infect Immun. 2006;74:4474‐4485. 16861633 10.1128/IAI.01924-05 PMC1539574

[23] Curtis MA , Percival RS , Devine D , et al. Temperature‐dependent modulation of *Porphyromonas gingivalis* lipid A structure and interaction with the innate host defenses. Infect Immun. 2011;79:1187‐1193. 21220483 10.1128/IAI.00900-10 PMC3067495

[24] Darveau RP , Pham TTT , Lemley K , et al. *Porphyromonas gingivalis* lipopolysaccharide contains multiple lipid a species that functionally interact with both toll‐like receptors 2 and 4. Infect Immun. 2004;72:5041‐5051. 15321997 10.1128/IAI.72.9.5041-5051.2004 PMC517442

[25] Herath TDK , Wang Y , Seneviratne CJ , et al. *Porphyromonas gingivalis* lipopolysaccharide lipid A heterogeneity differentially modulates the expression of IL‐6 and IL‐8 in human gingival fibroblasts. J Clin Periodontol. 2011;38:694‐701. 21752043 10.1111/j.1600-051X.2011.01741.x

[26] Noursadeghi M , Tsang J , Haustein T , Miller RF , Chain BM , Katz DR . Quantitative imaging assay for NF‐kappaB nuclear translocation in primary human macrophages. J Immunol Methods. 2008;329:194‐200. 18036607 10.1016/j.jim.2007.10.015 PMC2225449

[27] Kocgozlu L , Elkaim R , Tenenbaum H , Werner S . Variable cell responses to *P. gingivalis* lipopolysaccharide. J Dent Res. 2009;88:741‐745. 19734462 10.1177/0022034509341166

[28] Tang J , Wu T , Xiong J , et al. *Porphyromonas gingivalis* lipopolysaccharides regulate functions of bone marrow mesenchymal stem cells. Cell Prolif. 2015;48:239‐248. 25676907 10.1111/cpr.12173 PMC6496502

[29] Edwards CM , Mundy GR . Eph receptors and ephrin signaling pathways: a role in bone homeostasis. Int J Med Sci. 2008;5:263‐272. 18797510 10.7150/ijms.5.263 PMC2536716

[30] Hong JY , Shin MH , Chung KS , et al. EphA2 receptor signaling mediates inflammatory responses in lipopolysaccharide‐induced lung injury. Tuberc Respir Dis (Seoul). 2015;78:218‐226. 26175775 10.4046/trd.2015.78.3.218 PMC4499589

[31] Zhang Y , Wang XC , Bao XF , Hu M , Yu WX . Effects of *Porphyromonas gingivalis* lipopolysaccharide on osteoblast‐osteoclast bidirectional EphB4‐EphrinB2 signaling. Exp Ther Med. 2014;7:80‐84. 24348768 10.3892/etm.2013.1357 PMC3860981

[32] Pussinen PJ , Vilkuna‐Rautiainen T , Alfthan G , et al. Severe periodontitis enhances macrophage activation via increased serum lipopolysaccharide. Arterioscler Thromb Vasc Biol. 2004;24:2174‐2180. 15388525 10.1161/01.ATV.0000145979.82184.9f

[33] Jiang ZL , Cui YQ , Gao R , et al. Study of TNF‐alpha, IL‐1beta and LPS levels in the gingival crevicular fluid of a rat model of diabetes mellitus and periodontitis. Dis Markers. 2013;34:295‐304. 23478270 10.3233/DMA-130974 PMC3809972

[34] Kuroda C , Kubota S , Kawata K , et al. Distribution, gene expression, and functional role of EphA4 during ossification. Biochem Biophys Res Commun. 2008;374:22‐27. 18601903 10.1016/j.bbrc.2008.06.089

[35] Allan EH , Hausler KD , Wei T , et al. EphrinB2 regulation by PTH and PTHrP revealed by molecular profiling in differentiating osteoblasts. J Bone Miner Res. 2008;23:1170‐1181. 18627264 10.1359/jbmr.080324

[36] Tonna S , Takyar FM , Vrahnas C , et al. EphrinB2 signaling in osteoblasts promotes bone mineralization by preventing apoptosis. FASEB J. 2014;28:4482‐4496. 24982128 10.1096/fj.14-254300

[37] Tierney EG , McSorley K , Hastings CL , et al. High levels of ephrinB2 over‐expression increases the osteogenic differentiation of human mesenchymal stem cells and promotes enhanced cell mediated mineralisation in a polyethyleneimine‐ephrinB2 gene‐activated matrix. J Control Release. 2013;165:173‐182. 23201622 10.1016/j.jconrel.2012.11.013

[38] Zhang F , Zhang ZH , Sun D , Dong SW , Xu JZ , Dai F . EphB4 promotes osteogenesis of CTLA4‐modified bone marrow‐derived mesenchymal stem cells through cross talk with Wnt pathway in xenotransplantation. Tissue Eng Part A. 2015;21:2404‐2416. 26132739 10.1089/ten.TEA.2015.0012

[39] Myshkin E , Wang B . Chemometrical classification of ephrin ligands and Eph kinases using GRID/CPCA approach. J Chem Inf Comput Sci. 2003;43:1004‐1010. 12767159 10.1021/ci0256586

[40] Nagasawa T , Kiji M , Yashiro R , et al. Roles of receptor activator of nuclear factor‐kappaB ligand (RANKL) and osteoprotegerin in periodontal health and disease. Periodontol. 2000;2007:65‐84. 10.1111/j.1600-0757.2006.00185.x 17214836

[41] Bandow K , Maeda A , Kakimoto K , et al. Molecular mechanisms of the inhibitory effect of lipopolysaccharide (LPS) on osteoblast differentiation. Biochem Biophys Res Commun. 2010;402:755‐761. 21036155 10.1016/j.bbrc.2010.10.103

[42] Herath TD , Darveau RP , Seneviratne CJ , Wang CY , Wang Y , Jin L . Tetra‐ and penta‐acylated lipid A structures of *Porphyromonas gingivalis* LPS differentially activate TLR4‐mediated NF‐kappaB signal transduction cascade and immuno‐inflammatory response in human gingival fibroblasts. PLoS ONE. 2013;8:e58496. 23554896 10.1371/journal.pone.0058496 PMC3595299

[43] Ogawa T , Asai Y , Makimura Y , Tamai R . Chemical structure and immunobiological activity of *Porphyromonas gingivalis* lipid A. Front Biosci. 2007;12:3795‐3812. 17485340 10.2741/2353

